# Differences in Objective Physical Activity Between Children With Visual Impairment and Those With Normal Sight

**DOI:** 10.1167/tvst.13.7.18

**Published:** 2024-07-25

**Authors:** Srijana Adhikari, Fleur van Rens, Ruth M. A. van Nispen, Brook Galna, Ellen B. M. Elsman, Manish Poudel, Ger H. M. B. van Rens

**Affiliations:** 1 Tilganga Institute of Ophthalmology, Gaushala, Kathmandu, Nepal; 2 Department of Ophthalmology, Amsterdam UMC, Vrije Universiteit, Amsterdam, The Netherlands; 3 Quality of Care, Amsterdam Public Health, Amsterdam, The Netherlands; 4 School of Allied Health (Exercise Science) and Center for Healthy Aging, Murdoch University, Murdoch, Australia

**Keywords:** physical activity, visual impairment, accelerometry, children's vision, blindness

## Abstract

**Purpose:**

To compare objective physical activity (PA) levels of children with visual impairment (VI) and children with normal sight.

**Methods:**

One hundred children with VI and 100 age- and gender-matched normal-sighted peers 7 to 17 years of age wore an ActiGraph for 1 week. Activity count per minute (cpm) was modeled using a series of generalized linear mixed-effects models including vision, age, sex, time of day, and vision by time of day interaction. PA outcomes included mean counts per minute and proportion of time spent on sedentary, light, moderate, and vigorous intensity PA.

**Results:**

Data of 83 children with VI and 77 normal-sighted peers were included. Mean counts per minute were lower in children with VI (*P* < 0.001), especially during and after school. Children with VI were less sedentary (55%; 95% confidence interval [CI], 53–57) than children with normal sight before school (62%; 95% CI, 60–64) and over weekends: children with VI, 41% (95% CI, 39–43); children with normal sight, 45% (95% CI, 43–47). Yet, children with VI were more sedentary during school (36%; 95% CI, 34–37) compared with children with normal sight (30%; 95% CI, 29–32). They also spent more time performing light PA and less time performing moderate PA at school and vigorous PA across all periods of day (*P* < 0.001).

**Conclusions:**

Children with VI participated in light and moderate PA but did not perform as much vigorous PA as children with normal sight, especially during school hours.

**Translational Relevance:**

There is a need to promote more intense PA programs in schools tailored for children with VI.

## Introduction

Involvement in any kind of physical activity (PA) is beneficial for the physical and psychosocial development of children.^1^^–^^3^ Regular engagement in PA improves health and enhances physiological characteristics such as cardiovascular fitness, strength, endocrine function, and bone density.^4^ Greater PA is also correlated with better health-related quality of life in children.^5^ Despite these benefits, many children 5 to 17 years old do not participate in as much PA as recommended by the World Health Organization (WHO).^6^^–^^8^ Children with disabilities commonly experience barriers to engaging in PA^9^^,^^10^ and are less likely to be as physically active as their able-bodied peers and to meet the WHO recommended PA levels.^11^^,^^12^

Visual impairment (VI) is one of the leading causes of disability in children.^13^ There is scant research on the PA levels of children with VI. Studies using self-report questionnaires have shown that children and adolescents with VI report participating in less PA than recommended for their age.^14^^,^^15^ Self-report questionnaires are widely used to assess levels of PA; however, they have shown poor reliability and validity, especially in younger children.^16^^–^^18^ To overcome some of the challenges of self-reported PA in children, several studies have used accelerometry as an objective method of measuring PA and sedentary time. Accelerometers are lightweight, easy to use, and feasible devices to assess the PA levels of children.^19^^–^^21^ An example of an often-used accelerometer is the ActiGraph (Pensacola, FL).^22^^,^^23^ Very few studies have used accelerometry to quantify PA in children with VI.^24^^,^^25^ Houwen et al.^26^ reported lower intensity of PA in 48 children with blindness compared to 48 children with normal sight who were 6 to 12 years old, and overall levels of PA were low in both groups. They used the ActiGraph GT1M. Similarly, a study conducted by Al Jalal et al.^27^ compared PA in children with blindness, normal sight, and deafness and found that children with blindness were more sedentary compared to the other two groups. The accelerometer used in this study was the ActiGraph Bluetooth Smart wGT3X-BT.

However, most studies using accelerometry among children with VI have had several limitations including small sample sizes or no comparison groups.^28^^,^^29^ The few studies that have provided comparisons with normal-sighted peers^26^^,^^27^ included children at different schools or in different grade levels and living environments (e.g., living in a hostel). Furthermore, there has been a lack of consistency in the time frames captured by these studies. Some assessed PA during a particular time, such as during school hours or in a sports camp.^30^^–^^33^ Qi et al.^30^ studied the level of PA using actimeters worn by children with VI during different segments of the school day, such as during lunch, recess, or physical exercise classes, before and after school. This particular study found that children were more active on days with physical exercise than on the other days. However, the study did not have a comparison group of children with normal sight, and the study measured only combined moderate and vigorous PA.

Such limitations in these studies make it difficult to fully understand the PA of children with VI in relation to sighted peers. The aim of our study was to compare participants with regard to different intensities of PA levels across regular weeks and weekends using actimeters and to compare the intensity of PA between children with VI and normal sight of the same age, sex, and grade level of the same school. The integrated education system in Nepal makes it possible to recruit children with blindness and normal-sighted children from the same grade level. Furthermore, we aimed to explore levels of PA at different times of the school day and weekends. We hypothesized that PA participation and intensity would be lower in children with VI compared to children with normal sight across all times of the day. Knowing when during the day PA is mostly impacted by VI may help clinicians, researchers, families, and school administrators design appropriate interventions to facilitate PA in children with VI.

## Methods

This study is a part of the Nepal Pediatric Visual Impairment Study, which was carried out to investigate the causes of childhood VI and blindness, levels of participation, sleep behavior, and quality of life of life of children with a VI in Nepal, as well as their levels of PA. The study was approved by the Ethical Review Board of Nepal Health Research Council (proposal no. 239:2020). The study adhered to the tenets of the Declaration of Helsinki. Informed written consent was provided by the parents of the children, and consent was also provided by the children themselves.

### Design and Participants

In our cross-sectional comparative study, we recruited 100 children with VI who were 7 to 17 years old (primary school to grade 10) studying in the integrated schools for the blind in the Kathmandu Valley. These schools have an integrated system where children with VI follow the same curriculum and share the same classroom as children with normal sight. There were five schools selected in and around the Kathmandu Valley by purposive sampling. All children, with VI and with normal sight, follow dedicated physical education classes twice a week for 1 hour. Apart from this, children get a daily 1-hour lunch break in which they are allowed to play any type of sports activities. All five schools have large playgrounds with slides, seesaws, and fields to play various sports (e.g., basketball, volleyball, football), as well as space for indoor games such as table tennis. Because all schools adhere to a government curriculum, the same routine is followed by all of the schools. An equal number of children with normal sight (*n* = 100) of the same age, sex, school, and grade level were recruited as the control group. Exclusion criteria were developmental delay, cognitive impairment, or other disabilities apart from VI.

### Definitions of VI and Blindness

Vision loss was classified by the WHO categories of VI,^34^ where blindness is defined as best-corrected visual acuity (BCVA) < 3/60 in the better eye. Blindness was categorized into two groups: (1) BCVA < 3/60 to light perception (LP), and (2) no light perception (NLP). Normal sight was defined as having BCVA of 6/12 or better in the better seeing eye. Visual field was not assessed.

### Procedure

All children included in the study underwent ocular examinations by a pediatric ophthalmologist that included vision assessment, refraction, and anterior and posterior segment examination. The visual acuity of children with VI was assessed in each eye separately by using the Snellen chart, matching optotype chart, finger counting, or the use of torch light, depending on the level of visual acuity. Sociodemographic and clinical data collected were age, sex, living situation (hostel or boarding school vs. living with family), diagnosis, and visual acuity categories.

Children wore an ActiGraph wGT3X-BT activity monitor on the non-dominant wrist for 7 consecutive days. The ActiGraph is a small, lightweight triaxial device. Children were advised to wear the device all the time except during water-based activities (such as bathing). Children, parents, and caregivers were given verbal instructions on the use and care of the device. After 1 week, the device was retrieved from the children, and the stored data were downloaded using ActiLife 6.13.3 software. The downloaded data were exported as .csv files for further processing in MATLAB (MathWorks, Natick MA).

### Measurement of PA Levels

The ActiGraph device was set to record movement counts in 1-minute epoch intervals. The output of ActiGraph data was interpreted using cutoff points that define different intensities of PA. For this study, we used the Puyau et al.^35^ algorithm to classify the different intensities of PA identified for children. The cutoff points in this algorithm are (1) sedentary activity, <800 counts per minute (cpm); (2) light-intensity PA, 800 to 3199 cpm; (3) moderate PA, 3200 to 8199 cpm; and (4) vigorous PA, >8200 cpm. The following outcome variables were calculated for each child: (1) proportion of waking time spent sedentary; (2) proportion of waking time spent performing light, moderate, and vigorous intensity activity; and (3) PA intensity (mean counts per minute).

PA outcomes were calculated for three periods during school days (Sunday to Friday): before school, 4 AM to 9 AM; during school, 9 AM to 3 PM; and after school, 3 PM to 11 PM. No time increments were used for data obtained for Saturday, which represented PA during the weekend, as only Saturday is considered to be weekend in Nepal.

Wear time was calculated using the Troiano algorithm.^36^ Activity data for children who wore the device at least 4 days a week and 10 hours per day were included in the data analysis.^37^ In addition, the device had to be worn for at least 50% of the possible time within a period (e.g., at least 670 of the possible 1140 waking minutes for the weekend) for the data from that period to be included for analysis.

### Statistical Analysis

The normality of data and error residuals for each model were checked visually using histograms and residual plots. Descriptive statistics were calculated as means and standard deviations for continuous variables and frequencies for categorical variables. Also, 95% confidence intervals (CIs) were calculated to estimate precision of model coefficients. Differences in sociodemographic variables (such as age, gender, and living condition) between children with VI and normal sight were tested using independent samples *t*-tests or χ^2^ tests.

Generalized linear mixed-effects models were used to analyze the proportion of time that children engaged in sedentary, light, moderate, or vigorous intensity PA. More specifically, binomial distribution models (logit link with Nelder–Mead optimization) were fitted for each PA level using the lme4 R package (R Foundation for Statistical Computing, Vienna, Austria),^38^ the number of recorded 1-minute epochs were used as weights for each period of time. The proportion of time spent being active within each intensity category was modeled as a function of vision (normal sight or VI), sex (female or male), age (in years, centered around 12 years of age), and time of day (before school, during school, after school, or weekend) as fixed effects, and a random intercept was fitted for each child. We tested all vision-based two-way interactions between fixed effects in our preliminary analysis, which resulted in a vision by time of day interaction being added to the final model. The models are presented as odds ratios (change in odds from the reference value, or intercept). The reference (intercept) for the models was the estimated odds for a 12-year-old, normal-sighted female during school to participate in a particular intensity of activity (e.g., light-intensity PA) compared to another intensity of activity (e.g., sedentary, moderate, or vigorous activity).

Activity intensity (cpm) was modeled using a generalized linear mixed-effects model, with vision (normal sight or VI), sex (female or male), age (in years, centered around 12 years old), and time of day (before school, during school, after school, or weekend) entered as fixed effects and a random intercept was fit for each child. A gamma distribution with a log link and Nelder–Mead optimization was used to fit the model. Consistent with the models described earlier, we included a vision by time of day interaction. We present the model as percentage change from the reference value (intercept). The reference for this model was the estimated activity intensity for a 12-year-old, normal-sighted female during school.

To aid interpretation, we also calculated estimated marginal means for both sets of models, estimating the mean of each level of the fixed effects calculated at the mean of the remaining fixed effects (VI, age, sex, and time of day) using the emmeans package in R.^39^ Pairwise contrasts with Bonferroni adjustments for multiple comparisons were also conducted focusing on differences between normal-sighted and VI children for the vision by time of day interaction.

Preliminary analyses showed no significant differences between children with NLP and those with LP (*P* ≥ 0.05); therefore, all children with VI were consolidated into one VI group for the purposes of statistical modeling. We also compared the age, sex, and vision status of children whose data were missing compared to children whose data were included. This was to assess whether the missing data could bias our findings. Data entry was performed in Excel (Microsoft, Redmond, WA). Statistical analysis was performed in R 4.30. A threshold *P* < 0.05 (after Bonferroni adjustment for multiple comparisons, where applicable) was used to guide statistical interpretation.

## Results

### Demographic and Clinical Characteristics

Out of 200 participants, data for 160 participants were included in the analysis of PA. Among the 40 children who were excluded from analysis, 28 children did not fulfill the inclusion criteria (minimum wear time of 4 days per week and 10 hours per day), and activity data could not be downloaded for another 12 children due to mishandling of the ActiGraph. The data included consisted of 83 children with VI and 77 children with normal sight. There were no statistically significant differences between children with missing data in terms of age (*P* = 0.705), sex (*P* = 0.828), or visual acuity category (*P* = 0.289). Children with VI were more likely to reside in a hostel or boarding school compared to their normal-sighted peers (*P* < 0.001) and were slightly older despite our matching efforts (Table 1).

**Table 1.** Characteristics of Children With and Without VI

Characteristics	Normal-Sighted Children (*n* = 77)	Children With VI (*n* = 83)	*P*
Age (y), mean ± SD	12.0 ± 2.9	13.1 ± 2.3	0.03
Female, *n* (%)	32 (42)	31 (37)	0.58
Vision category, *n* (%)			—
Normal (>6/12)	77 (100)	—	
VI (<3/60, LP)	—	74 (89)	
VI (NLP)	—	9 (11)	
Living in a hostel, *n* (%)	7 (9)	75 (90)	<0.001
Etiology of VI, *n*			
**Whole globe**	—	**38**	—
Phthisis bulbi	—	13	
Anophthalmous	—	6	
Microphthalmous	—	15	
Buphthalmous	—	4	
**Cornea**	—	**15**	
Keratoconus	—	7	
Corneal opacity	—	0	
Anterior staphyloma	—	7	
Microcornea	—	1	
**Uvea**	—	**1**	
Burnt-out uveitis	—	1	
**Lens**	—	**6**	
Cataract	—	2	
Aphakia with amblyopia	—	4	
Pseudophakia with amblyopia	—	0	
Subluxated lens	—	0	
**Retina**	—	**32**	
Retinal dystrophy	—	15	
Albinism	—	2	
Pathological myopia	—	0	
Retinopathy of prematurity sequalae	—	9	
Retinal detachment	—	2	
Choroidal coloboma	—	0	
Macular scar	—	4	
**Optic nerve**	—	**8**	
Optic atrophy	—	6	
Optic nerve hypoplasia	—	2	

### Categorical Levels of PA

The mixed-effects binomial regression showed that older age was associated with a greater proportion of time being sedentary or performing light PA and a smaller amount of time performing moderate or vigorous PA. It also showed that boys spent more time being sedentary and a smaller amount of time performing moderate PA compared to girls (Table 2). Comparison of estimated marginal means indicated that children with VI spent a similar proportion of their time being sedentary (Table 3). Children with VI spent more time performing light PA but less time performing moderate and vigorous PA than their normal-sighted peers. Post hoc contrasts for the vision by time of day interactions showed that children with VI were less sedentary than normal-sighted children before school and during the weekend, yet were more sedentary during school (Fig.). Children with VI also spent more time performing light PA across all periods of the day than their normal-sighted peers. However, children with VI spent less time performing moderate PA at school and vigorous PA during all periods of the day compared to children with normal sight, especially during school.

**Table 2.** Binomial (Logit) Models for the Proportion of Time Children Performed Different Levels of PA

Predictors	Sedentary, OR (95% CI), *P*	Light PA, OR (95% CI), *P*	Moderate PA, OR (95% CI), *P*	Vigorous PA, OR (95% CI), *P*
Intercept				
Reference: 12-year-old, normal-sighted female during school	0.24 (0.21–0.26), *P* < 0.001	0.47 (0.43–0.50), *P* < 0.001	0.60 (0.55–0.66), *P* < 0.001	0.115 (0.10–0.14), *P* < 0.001
Visual impairment	1.64 (1.47–1.83), *P* < 0.001	1.19 (1.10–1.30), *P* < 0.001	0.75 (0.68–0.83), *P* < 0.001	0.31 (0.26–0.4), *P* < 0.001
Age, ≥12 y	1.03 (1.01–1.05), *P* < 0.001	1.00 (1.01–1.03), *P* = 0.007	0.96 (0.94–0.97), *P* < 0.001	0.92 (0.89–0.95), *P* < 0.001
Male	1.19 (1.06–1.32), *P* = 0.003	0.97 (0.90–1.04), *P* = 0.396	0.85 (0.77–0.94), *P* = 0.002	0.86 (0.70–1.06), *P* = 0.170
Time of day				
Before school	6.20 (6.10–6.31), *P* < 0.001	0.42 (0.42–0.43), *P* < 0.001	0.37 (0.36–0.38), *P* < 0.001	0.362 (0.35–0.37), *P* < 0.001
After school	2.43 (2.4–2.47), *P* < 0.001	0.71 (0.70–0.72), *P* < 0.001	0.65 (0.64–0.66), *P* < 0.001	0.8 (0.78–0.81), *P* < 0.001
Weekend	3.07 (3.02–3.13), *P* < 0.001	0.60 (0.59–0.62), *P* < 0.001	0.59 (0.58–0.60), *P* < 0.001	0.731 (0.71–0.76), *P* < 0.001
Vision × time of day interaction				
Visual impairment × before school	0.45 (0.44–0.46), *P* < 0.001	1.32 (1.29–1.35), *P* < 0.001	1.427 (1.39–1.463), *P* < 0.001	1.784 (1.69–1.9), *P* < 0.001
Visual impairment × after school	0.62 (0.61–0.63), *P* < 0.001	1.07 (1.05–1.1), *P* < 0.001	1.259 (1.23–1.28), *P* < 0.001	1.391 (1.33–1.45), *P* < 0.001
Visual impairment × weekend	0.52 (0.51–0.54), *P* < 0.001	1.20 (1.17–1.23), *P* < 0.001	1.34 (1.31–1.38), *P* < 0.001	1.57 (1.497–1.65), *P* < 0.001
Marginal *R*^2^	8.1%	2.8%	3.3%	8.8%
Conditional *R*^2^	11.3%	4.5%	6.1%	19.0%

**Table 3.** Estimated Marginal Means for the Proportion of Time Children Performed Different Levels of PA

Predictors	Predictor Level	Sedentary (%), Mean (95% CI)	Light PA (%), Mean (95% CI)	Moderate PA (%), Mean (95% CI)	Vigorous PA (%), Mean (95% CI)
Vision	Normal vision	40.7 (38.8–42.6)	**23.4 (22.4–24.4)**	25.1 (23.8–26.5)	**6.5 (5.7–7.5)**
	VI	41.2 (39.4–43.1)	**29.3 (28.2–30.4)**	23.9 (22.6–25.2)	**3.0 (2.6–3.5)**
Age^a^	9 y	37.9 (35.9–40.0)	25.0 (23.9–26.2)	27.3 (25.8–28.9)	5.8 (5.0–6.7)
	12 y	40.5 (39.1–41.8)	26.0 (25.3–26.8)	24.9 (24.0–25.9)	4.7 (4.2–5.1)
	15 y	43.0 (41.3–44.8)	27.0 (26.1–28.0)	22.7 (21.5–23.8)	3.7 (3.3–4.2)
Sex	Female	**39.0 (36.9–41.0)**	26.5 (25.4–27.7)	**26.0 (24.5–27.5)**	4.8 (4.1–5.6)
	Male	**43.0 (41.3–44.7)**	25.9 (25.0–26.8)	**23.1 (21.9–24.2)**	4.2 (3.7–4.7)
Time of day	Before school	**58.5 (57.1–59.9)**	**19.9 (19.3–20.6)**	**17.3 (16.6–18.1)**	**2.7 (2.5–3.0)**
	During school	**25.2 (24.2–26.3)**	**33.7 (32.8–34.6)**	**32.1 (31.0–33.2)**	**5.5 (5.0–6.0)**
	After school	**39.3 (38.0–40.6)**	**27.3 (26.5–28.1)**	**25.5 (24.7–26.6)**	**5.2 (4.7–5.7)** ^b^
	Weekend	**42.9 (41.6–44.3)**	**25.1 (24.4–25.9)**	**24.4 (23.5–25.4)**	**5.1 (4.6–5.6)** ^b^
Vision by time of day interaction	Before school				
	Normal vision	**62.0 (60.1–63.9)**	**16.6 (15.8–17.4)**	16.9 (15.9–17.9)	**3.6 (3.1–4.1)**
	VI	**55.0 (53.0–56.9)**	**23.8 (22.8–24.8)**	17.8 (16.8–18.9)	**2.1 (1.8–2.4)**
	During school				
	Normal vision	**20.8 (19.5–22.2)**	**31.8 (30.6–33.0)**	**35.3 (33.7–37.0)**	**9.3 (8.1–10.6)**
	VI	**30.2 (28.6–31.9)**	**35.6 (34.4–36.9)**	**29.0 (27.6–30.5)**	**3.2 (2.7–3.6)**
	After school				
	Normal vision	39.0 (37.1–40.9)	**24.9 (23.9–26.0)**	26.2 (24.8–27.6)	**7.6 (6.6–8.6)**
	VI	39.5 (37.7–41.4)	**29.8 (28.7–30.9)**	25.1 (23.8–26.4)	**3.5 (3.0–4.0)**
	Weekend				
	Normal vision	**44.7 (42.7–46.7)**	**22.0 (21.0–22.9)**	24.3 (23.0–25.7)	**7.0 (6.1–8.0)**
	VI	**41.2 (39.3–43.1)**	**28.6 (27.5–29.8)**	24.5 (23.2–25.8)	**3.7 (3.2–4.2)**

Bold type indicates significant post hoc contrasts between predictor levels (*P* < 0.05) after Bonferroni adjustments for multiple comparisons.

a No post hoc contrasts were performed for age, as it is a continuous variable and estimates for each age are shown to aid interpretation.

b After school versus weekend contrast (*P* > 0.05).

**Figure.**
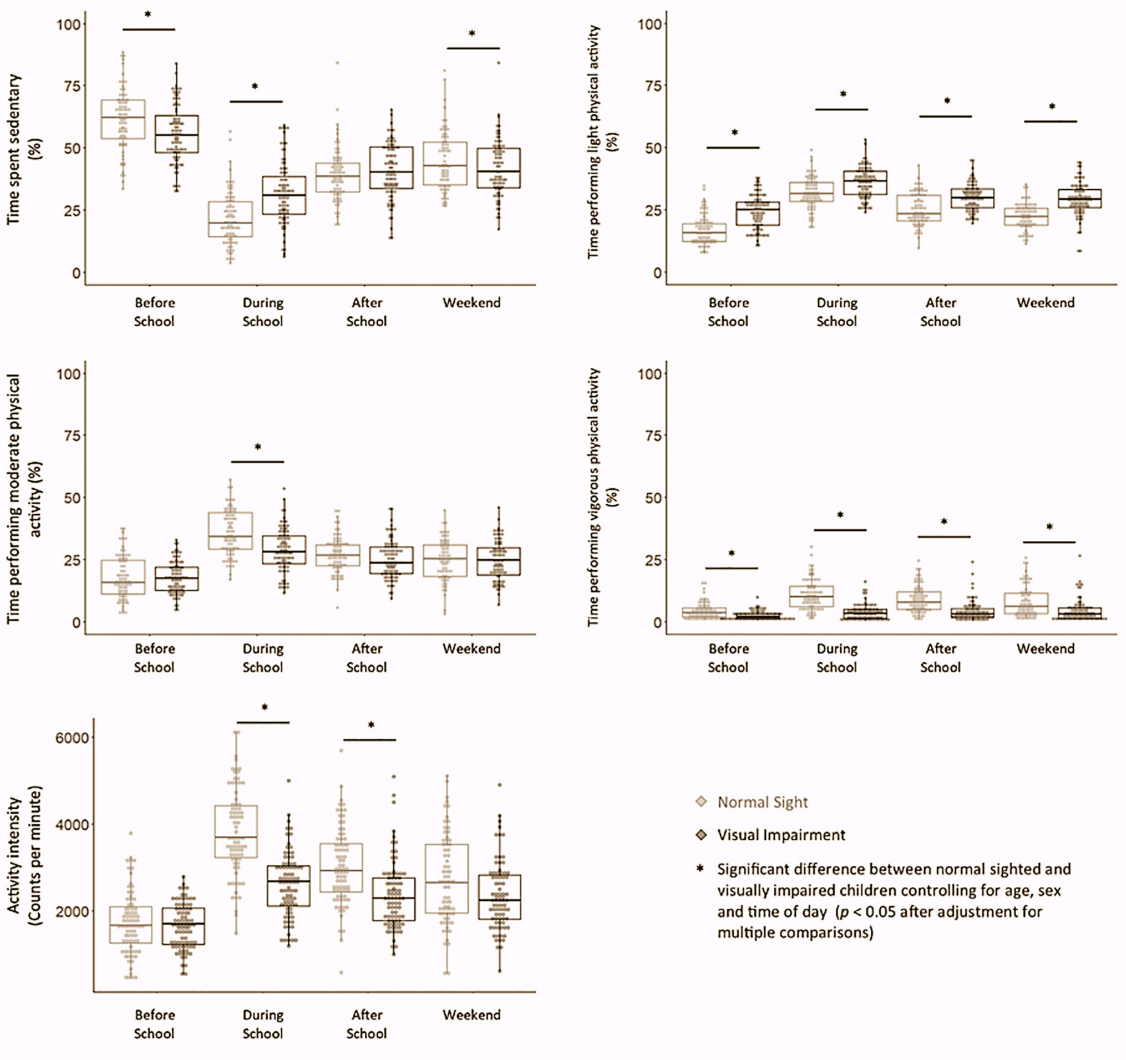
Boxplots illustrating the time that normal-sighted children (light gray) and visually impaired children (dark gray) spent performing various levels of physical activity and their mean activity intensity for different portions of the day.

### Intensity of Activity

Older age and male sex were associated with lower activity intensity (Tables 4 and 5). Comparison of estimated marginal means showed that children with VI moved less intensely, as indicated by a lower mean rate of ActiGraph activity counts per minute (Table 5). Post hoc contrasts for the vision by time of day interaction showed that children with VI moved with less intensity (i.e., lower mean activity counts per minute) compared to normal-sighted peers during school and after school, but with a similar intensity before school and on the weekend (Table 5, Fig.). Even though children with VI participated in less vigorous PA than their normal-sighted peers, on average, the Figure clearly shows that some children with VI performed more vigorous PA than a majority of normal-sighted children.

**Table 4.** Gamma Regression of Mean Activity Counts Per Minute

Predictors	Mean Activity Counts Per Minute, Percent (%) of Reference (95% CI), *P*
Intercept: Reference 12-year-old, normal-sighted female during school	3999 (3590–4454), *P* < 0.001
Vision: VI	69.0 (61.0–78.1), *P* < 0.001
Age: ≥12 years	97.2 (95.5–99.0), *P* = 0.002
Male sex	89.7 (80.7–99.8), *P* = 0.046
Time of day	
Before school	44.0 (40.2–47.5), *P* < 0.001
After school	77.0 (71.3–83.1), *P* < 0.001
Weekend	64.8 (63.2–74.1), *P* < 0.001
Vision × time of day interaction	
Visual impairment × before school	145.7 (130.9–168.0), *P* < 0.001
Visual impairment × after school	117.3 (105.5–130.5), *P* = 0.003
Visual impairment × weekend	129.1 (115.8–144.0), *P* = 0.001
Marginal *R*^2^	44.7%
Conditional *R*^2^	62.6%

**Table 5.** Estimated Marginal Means for Activity Intensity

Predictor	Predictor Level	Activity Counts Per Minute, Mean (95% CI)
Vision	Normal vision	**2** **586 (2** **397** **–** **2** **789)**
	VI	**2** **176 (2** **022** **–** **2** **342)**
Age	9 y	2592 (2392–2836)
	12 y	2392 (2253–2540)
	15 y	2208 (2039–2368)
Sex	Female	**2** **504 (2** **306** **–** **2** **719)**
	Male	**2** **247 (2** **102** **–** **2** **401)**
Time of day	Before school	**1** **644 (1** **545** **–** **1** **750)**
	During school	**3** **097 (2** **910** **–** **3** **295)**
	After school	**2** **582 (2** **426** **–** **2** **747)** ^a^
	Weekend	**2** **408 (2** **261** **–** **2** **565)** ^a^
Vision by time of day interaction	Before school	
	Normal vision	1639 (1499–1792)
	VI	1649 (1513–1798)
	During school	
	Normal vision	**3** **727 (3** **410** **–** **4** **074)**
	VI	**2** **573 (2** **361** **–** **2** **805)**
	After school	
	Normal vision	**2** **868 (2** **624** **–** **3** **135)**
	VI	**2** **323 (2** **132** **–** **2** **533)**
	Weekend	
	Normal vision	2550 (2329–2793)
	VI	2274 (2084–2481)

Bold type indicates significant post hoc contrasts between predictor levels (*P* < 0.05) after Bonferroni adjustments for multiple comparisons.

a After school versus weekend contrast (*P* > 0.05).

## Discussion

The aim of our study was to use actimeters to compare PA levels of children with VI in Nepal with their age- and sex-matched normal-sighted peers attending the same school at the same grade level. Our findings indicate that children with VI had lower overall PA levels compared to their normal-sighted peers. This finding is in line with research by Houwen et al.^26^ and Kozub et al.^29^ but contrary to the findings by Demirturk et al.^33^ and Smith et al.^40^ However, their studies were respectively based on subjective questionnaires (Demirturk et al.^33^) and included children with less severe VI (VA 6/12 or less) (Smith et al.^40^) compared to the present study (VA less than 3/60). We speculate that the lower level of PA in children with VI could be due to environmental barriers such as lack of accessible playgrounds, reduced motivation, or difficulty in navigation.

We found that children in both groups spent the largest proportion of time (∼40%) in sedentary activity. This is similar to findings by Houwen et al.^26^ and aligns with the increasing trend of sedentary behavior among children.^41^^,^^42^ Further, we found that children with VI spent more time in light PA and less time engaging in moderate and vigorous PA than their normal-sighted peers. This finding is again similar to those of Houwen et al.,^26^ who also compared activity levels in VI and normal-sighted children using the ActiGraph. Their study included children with moderate VI (less than 6/18 to 6/60) and severe VI (less than 6/60). This indicates that children with VI are more involved in light PA, such as walking, preparing food, and getting ready for school. Children, both sighted and VI, who reside in hostels in Nepalese schools perform morning routines themselves; however, in Nepalese households, the parents do the chores for their children and prepare them for school.

Although there was no statistically significant difference between the groups for moderate PA, normal-sighted children were more involved in vigorous PA than children with VI. This suggests that children with VI are less likely to be involved in vigorous sports and activities such as football, basketball, and cycling, even though these activities were available for all children, including those with VI. We did not measure the visual field of children; however, the most common etiology of VI in our study was whole globe anomalies followed by retinal diseases. Both of these often involve visual field loss and might have contributed to less involvement of children with VI in PA. Also, our findings support those who have previously outlined the additional barriers children with VI face when trying to participate in vigorous PA^43^^,^^44^ and further highlight the urgent need for more accessible means for children with VI to engage in vigorous PA.

Encouragingly, some children with VI were more involved in vigorous PA than most normal-sighted children. This finding suggests that vigorous PA is possible for this group of children and highlights the need for further research on the types of activities that these children are involved with and the accessibility to these activities. This would help tailor PA for children with VI.

Unlike previous studies,^45^^,^^46^ our study presents novel findings regarding the proportion of time that children are engaged in different levels of PA at various times during the week and on weekends, including children with VI and their normal-sighted peers. Our study findings indicate that, before school, children with VI were more involved in light-intensity PA and less in sedentary PA compared to normal-sighted children. No significant differences in the mean PA or in the proportion of time spent in sedentary or moderate PA were found. Because most children with VI reside in residential schools, this finding may be explained by their participation in morning routine activities such as cleaning dishes, getting dressed, and preparing for school by themselves, all of which involve light levels of PA. This interpretation highlights the possible role that living arrangements may play in levels of PA among children with VI and may also help explain why light-intensity PA may be greater among children with VI than their normal-sighted peers.

During school hours, children with normal sight were more physically active than children with VI; they engaged in more moderate and vigorous PA, and children with VI spent longer engaged in sedentary and light PA. This finding suggests that the activities available at school may not always be accessible to children with VI compared to normal-sighted children.

There may be various factors associated with the inaccessibility of PA for children with VI, such as a lack of vigorous sports activities suitable for children with VI, including gymnastics, swimming, dancing, and physical training. Additionally, fewer opportunities to participate, unavailability of trained teachers in school, low levels of self-esteem and motivation, and lack of a favorable physical environment may also play a role.^47^^–^^50^ Similarly, various other factors may also be related to the school environment and education system in Nepalese schools. Children with VI are in the same classrooms and follow the same curriculum as normal-sighted children. The only difference is that students with VI read and write using braille and the sighted children read and write normal text. Special assistants are available for children with VI if they need extra attention regarding the curriculum. Otherwise, physical education and extracurricular activities are the same for all children. More research is needed to determine the relative importance of these and other factors. In their review, Elsman et al.^51^ found that sports camps, among other factors, might be helpful in improving PA in children with VI, a finding similar to that of a study by Qi et al.,^30^ who found that children were more physically active on days of physical education classes in school than on other days. Consequently, the PA levels of children with VI during school time could potentially be improved by integrating accessible physical education classes or games into the school curriculum or during lunch time.

During the weekend, which includes only Saturday in Nepal, children were sedentary approximately 43% of their waking day, and there was no statistically significant difference between children with VI and normal-sighted children in any of the PA levels. This indicates that the children are possibly involved in similar types of recreational activities, such as watching television or using their mobile devices, or spending time in indoor activities with their families or friends in hostel during the weekend. Another important finding of our study was that younger age was associated with spending more time performing PA and performing more intense activity in both sighted and children with VI, which is supported by the findings of other studies regarding children with VI.^28^^,^^29^ Hence, the findings from our study are slightly different than our hypothesis that children with VI are less physically active at all times of the day than their normal-sighted peers.

Strengths of our study include a relatively large sample of children with VI, a control group of normal-sighted peers, and an analysis of PA during different times of the day, both during the week and on the weekend. Moreover, because of the inclusive education system in Nepal, we were able to match children with VI to their direct peers as the comparison group who belonged to the same class and grade level as children with VI.

There were some limitations in our study. First, each child wore an ActiGraph on the wrist, even though the algorithm that we used in this study (Puyau et al.^35^) was developed using data collected from actimeters attached to the hip. We opted for the wrist-worn ActiGraph to enable data collection for the overall project, which included collecting sleep parameter data from these children, as well. Better compliance for wrist-worn compared to hip-worn devices has been reported in children and adolescents.^52^ However, it is likely that we overestimated the PA levels of children because of the wrist placement. Arm movements may be more frequent than hip movements and are interpreted as steps when the device is placed on the wrist. There are a few studies that have reported greater accuracy with hip placement than with wrist placement,^53^^–^^55^ but a study by Staudenmayer et al.^56^ showed better accuracy with wrist-worn devices. Because of the wrist placement, it is likely that we overestimated the PA levels of children. Nevertheless, the accuracy of such devices must be confirmed by data obtained under free-living conditions.^37^ To address this, we analyzed the data using the mean activity intensity, which does not rely on thresholds and found the same results. Further validation of PA algorithms for wrist-worn devices is warranted for children.

Another limitation is missing data due to poor wear compliance, poor handling of the ActiGraph device, or technological difficulties, common with this type of research in children. Finally, due to the small sample size of children with NLP we did not compare results from sighted children with those with those for LP and NLP separately in the full statistical analysis. Although our preliminary analysis suggested no statistically significant differences in PA between children with LP and NLP, it is possible that children with NLP are less physically active than children with LP. As such, we recommend purposeful recruitment of groups of children with NLP in future studies.

Our study provides novel insights into the PA of children with VI at different times of the week. Based on our findings, we encourage policymakers to explore ways to help children with VI to be more physically active. In particular, there is a need to promote PA interventions tailored to the specific needs of children with VI, especially those that could help increase participation in vigorous PA in schools. To aid in the development of these targeted interventions, further studies are necessary to identify barriers to PA for children with VI and identify the resources and information schools require to facilitate PA for VI children.
